# Trypanosome mRNA recapping is triggered by hypermethylation originating from cap 4

**DOI:** 10.1093/nar/gkae614

**Published:** 2024-07-16

**Authors:** Anna V Ignatochkina, Jesavel A Iguchi, Anilkumar R Kore, C Kiong Ho

**Affiliations:** Department of Infection Biology, Graduate School of Comprehensive Human Sciences, Institute of Medicine, University of Tsukuba, Ibaraki 305-8575, Japan; Department of Infection Biology, Graduate School of Comprehensive Human Sciences, Institute of Medicine, University of Tsukuba, Ibaraki 305-8575, Japan; Life Sciences Solutions Group, Thermo Fisher Scientific, 2130 Woodward Street, Austin, TX 78744-1832, USA; Department of Infection Biology, Graduate School of Comprehensive Human Sciences, Institute of Medicine, University of Tsukuba, Ibaraki 305-8575, Japan

## Abstract

RNA methylation adjacent to the 5′ cap plays a critical role in controlling mRNA stability and protein synthesis. In trypanosomes the 5′-terminus of mRNA is protected by hypermethylated cap 4. Trypanosomes encode a cytoplasmic recapping enzyme TbCe1 which possesses an RNA kinase and guanylyltransferase activities that can convert decapped 5′-monophosphate-terminated pRNA into GpppRNA. Here, we demonstrated that the RNA kinase activity is stimulated by two orders of magnitude on a hypermethylated pRNA derived from cap 4. The *N6, N6*-*2′-O* trimethyladenosine modification on the first nucleotide was primarily accountable for enhancing both the RNA kinase and the guanylyltransferase activity of TbCe1. In contrast, *N6* methyladenosine severely inhibits the guanylyltransferase activity of the mammalian capping enzyme. Furthermore, we showed that TbCmt1 cap (guanine *N7*) methyltransferase was localized in the cytoplasm, and its activity was also stimulated by hypermethylation at 2′-*O* ribose, suggesting that TbCe1 and TbCmt1 act together as a recapping enzyme to regenerate translatable mRNA from decapped mRNA. Our result establishes the functional role of cap 4 hypermethylation in recruitment and activation of mRNA recapping pathway. Methylation status at the 5′-end of transcripts could serve as a chemical landmark to selectively regulate the level of functional mRNA by recapping enzymes.

## Graphical Abstract

**Graphical Abstract:**
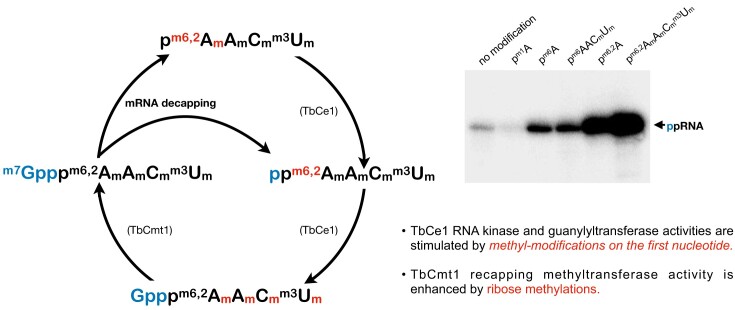

## Introduction

The *N7*-methylguanosine cap (^m7^G or cap 0) of the eukaryotic mRNAs is formed in the nucleus to protect mRNA from degradation and promote translation initiation. RNA modifications, particularly m6A modification adjacent to cap 0, have gained significant attention due to their involvement in various biological processes and their association with numerous diseases. In most eukaryotic species, the first and second nucleotides adjacent to the cap 0 are frequently methylated at 2′-*O* ribose (1,2). The majority of the transcripts initiated by adenine are further methylated by PCIF1 (or CAPAM) at the *N6*-position to form ^m7^Gppp^m6^A_m_ (3–8). These modifications adjacent to the cap structure can alter mRNA stability and affect translation efficiency (5–7,9,10) and could play a pivotal role for viral mRNA in evading recognition by the host immune system (11–13). The m6A methylation on ^m7^Gppp^m6^A_m_ could be demethylated by Fat mass and obesity-associated protein (FTO), implying that the fate of mRNA is dynamically regulated by cap methylation (9).

The most extensive modification at the 5′-end of mRNA is present in trypanosomes and other related kinetoplast protozoans, called cap 4. Cap 4 consists of a ^m7^G cap with *N6,N6*-*2′-O* trimethylation on the first adenine (^m6,2^A_m_), *2′-O* methylations on the second adenine (A_m_) and third cytosine (C_m_), and *N3-2′-O* dimethylation on the fourth uracil (^m3^U_m_) (14). Cap 4 is formed on a spliced leader (SL) RNA, which is transferred to pre-mRNA by trans-splicing (15,16). Earlier studies using AdoHcy and sinefungin, inhibitors for S-adenosyl-L-methionine dependent methylation reaction, suggest hypermethylation on the SL RNA is required for trans-splicing (17,18). However, these inhibitors could also prevent guanine *N7* methylation on the ^m7^G cap, which in turn was required for cap 4 hypermethylation on the SL-RNA (19,20). Cap-dependent RNA methyltransferases responsible for 2′-O ribose methylations at position 1 (TbMTr1), 2 (TbMTr2 or TbCom2) and 3 and 4 (TbMTr3/4) were identified and characterized in *T. brucei* (21–24). Genetic knockouts or RNAi knockdowns of individual cap 4 methyltransferases did not show a significant impact on mRNA level or translation efficiency and were viable (24–26), suggesting that fully methylated cap 4 is not required for trans-splicing. However, the double knockouts of TbMTr1 with either TbMTr2 or TbMTr3 cannot be established. RNA methyltransferase responsible for m6A methylation on the first adenine (PCIF1) that can modify ^m7^GpppA_m_ RNA to ^m7^Gppp^m6^A_m_ RNA has been identified in *T. cruzi* (7). Cap-dependent RNA methyltransferase activities responsible for *N6,N6* dimethyladenosine and *N3-*methyluridine have not yet been reported.

Trypanosomes encode two sets of ^m7^G capping enzymes, one in the nucleus and another in the cytoplasm. The nuclear capping enzyme, TbCgm1, possesses guanylyltransferase and *N7* guanine RNA methyltransferase activities which can convert diphosphate-terminated RNA (ppRNA) into ^m7^Gppp-terminated RNA (19,20). Depletion of TbCgm1 results in the accumulation of uncapped SL RNA and blocks subsequent methylation events that lead to cap 4 formation, suggesting that TbCgm1 is responsible for SL RNA capping. TbCgm1 likely acts with TbCet1 RNA triphosphatase, which converts triphosphate-terminated RNA into ppRNA, a substrate for TbCgm1 (27–29).

As a part of mRNA turnover process, cap can be removed by decapping enzyme, and the remaining 5′-phosphorylated RNA (pRNA) is thought to be rapidly degraded by a 5′-to-3′ exonuclease (Xrn1/Rat1) (30,31). However, the uncapped transcripts are present in the cells and could later become translationally active by reacquiring the cap in the cytoplasm (32,33). The cytoplasmic capping enzyme TbCe1 possesses RNA kinase and guanylyltransferase activities that can convert pRNA into GpppRNA via the formation of ppRNA intermediate (34). Depletion of TbCe1 results in accumulation of pRNA and, therefore, has been proposed to function to regenerate translatable mRNA by converting decapped pRNA into capped mRNA.

Here, we report the functional role of cap 4 methylation in the cytoplasmic mRNA recapping pathway in trypanosomes. We demonstrate that hypermethylation on the uncapped RNA can enhance TbCe1 activities for recapping. We also show that TbCmt1 cap (guanine *N7*) RNA methyltransferase is localized in the cytoplasm, and its activity is also upregulated by hypermethylation. Furthermore, our study indicates that cap methylation could potentially regulate the mRNA recapping pathway in mammalian cells.

## Materials and methods

### Recombinant enzymes, protein expression and purification

Recombinant TbCe1 was expressed as a His-Smt3 TbCe1 fusion protein in *E. coli* and purified by Ni-NTA agarose as described (34). Upon removal of the N-terminal His-Smt3 tag by Ulp1 protease, TbCe1 protein was further purified on 15 - 30% glycerol gradient sedimentation and a peak fraction was used to assay for enzymatic activity. His-tagged TbCmt1 and His-tagged Mce1 were produced in *Escherichia coli* and purified from soluble bacterial extracts using Ni-agarose chromatography as described previously (35,36). The final protein concentrations were determined with the Bio-Rad dye reagent using bovine serum albumin as the standard and enzymes were stored at –80ºC. Vaccinia virus capping enzyme was purchased from New England Biolabs (10,000 units/ml, Lot #10182558).

### Synthetic RNA oligonucleotides

RNA oligonucleotides used in this study are shown in Table 1. All synthetic oligoribonucleotides possess 5′-phosphate with identical 21-mer sequence, AACUAACGCUAUUAUUAGAAC, which corresponds to the first 21 nucleotides of *T. brucei* SL RNA (34). The p^m6,2^A_m_A_m_C_m_^m3^U_m_ RNA were synthesized as described (37). All RNAs were purified using HPLC and were verified with mass-spectrometry analysis. The concentration and purity of the oligoribonucleotides were confirmed with optical density and by PAGE stained with GelRed (Biotium, USA). The presence of 2′-O ribose methylations was confirmed by RNase (RNase A, T1 and T2) digestion of a ^32^P-cap labeled RNA (Supplementary Figure S1).

**Table 1.** List of synthetic RNA oligonucleotides

Abbreviations	Modifications	Sources
p^m6,2^A_m_A_m_C_m_^m3^U_m_	*N6*, *N6*, 2′-*O* trimethyladenosine on A1 2′-*O* ribose methylations on A2 and C3 *N3-*2′-*O*-dimethyluridine on U4	ThermoFisher Scientific (Austin, TX, USA)
p^m6,2^A_m_	*N6*, *N6*, 2′-*O* trimethyladenosine on A1	ThermoFisher Scientific (Austin, TX, USA)
p^m6,2^A_m_A_m_C_m_U_m_	*N6*, *N6*, 2′-*O* trimethyladenosine on A1 2′-*O* ribose methylations on A2, C3 and U4	ThermoFisher Scientific (Austin, TX, USA)
p^m6,2^A	*N6*, *N6* dimethyladenosine on A1	ThermoFisher Scientific (Austin, TX, USA)
p^m6^AAC_m_U_m_	*N6* methyladenosine on A1 2′-*O* ribose methylation on C3 and U4	GeneDesign Inc /Ajinomoto (Japan)
p^m6^A	*N6* methyladenosine on A1	GeneDesign Inc /Ajinomoto (Japan)
pA_m_A_m_C_m_^m3^U_m_	2′-*O* ribose methylations on A1, A2 and C3 *N3*, 2′-O-dimethyluridine on U4	ThermoFisher Scientific (Austin, TX, USA)
pA_m_A_m_C_m_U_m_	2′-*O* ribose methylations on A1, A2, C3 and U4	Bio-Synthesis Inc. (Lewisville, TX, USA)
pA_m_	2′-*O* ribose methylation on A1	Bio-Synthesis Inc. (Lewisville, TX, USA)
p^m1^A	*N1*-methyladenosine on A1	Bio-Synthesis Inc. (Lewisville, TX, USA)
pRNA or unmodified	No modification	Bio-Synthesis Inc. (Lewisville, TX, USA)

All oligoribonucleotides are phosphorylated at 5′-end with a sequence of 5′-AACUAACGCUAUUAUUAGAAC-3′. A1: adenine at position 1. A2: adenine at position 2, C3: cytosine at position 3, and U4: uracil at position 4.

### Preparation of diphosphate-terminated RNA (ppRNA) and guanylated RNA (GpppRNA) substrates from modified pRNAs

Modified and unmodified ^32^P-labeled diphosphate-terminated RNAs (**p**pRNA; boldface indicates labeled phosphate) were prepared by treating the 5′-monophosphate terminated RNA oligonucleotides (Table 1) with TbCe1 and [**γ**-^32^P] ATP. Reaction mixtures (50 μl) containing 50 mM Tris–HCl (pH 8.0), 2 mM MgCl_2_, 1 mM dithiothreitol, 8 units of RNase Inhibitor (Nacalai Tesque Inc., 40 u/μl), 10 μM of [**γ**-^32^P] ATP, 0.2 – 0.5 nmol of pRNA oligonucleotide were incubated at 27 ºC for 60 min with 2 μg of TbCe1 (K288A) protein, an active site mutant form of TbCe1 that lacks guanylyltransferase activity to prevent formation of GpppRNA (34). For preparation of [^32^P]-labeled G**p**ppRNA, 2 nmol of either modified and unmodified pRNA oligonucleotide was incubated in a reaction mixture (50 μl) containing 50 mM Tris–HCl (pH 8.0), 2 mM MgCl_2_, 1 mM dithiothreitol, 175 units of RNase Inhibitor, 100 μM of ATP, 20 μM [α-^32^P] GTP with 15 μg of wild-type TbCe1 at 27ºC for 15 min. Radiolabeled **p**pRNAs and G**p**ppRNAs were purified using native 15 - 18% PAGE, visualized on PhosphorImager, and cut and eluted with TE. Recovery of labeled RNA was assessed by scintillation counting. The molar concentrations of **p**pRNA and G**p**ppRNA were calculated according to the specific activity of the input [γ-^32^P] ATP and [α-^32^P] GTP donor in the reaction, respectively.

### RNA kinase assay

Standard reaction (10 μl) contained 50 mM Tris–HCl (pH 8.5), 1 mM dithiothreitol, 0.5 mM MgCl_2_, 100 nM of pRNA substrate. [**γ**-^32^P] ATP and TbCe1 as specified were incubated at 27°C. The reactions were quenched by the addition of 10 μl of 90% formamide, EDTA (20 mM). The samples were analyzed using 18% PAGE containing 7 M urea in 45 mM Tris-borate, EDTA (1 mM). The products were visualized and quantified by scanning the gel with a Fujix BAS-2500 PhosphorImager.

### Cap methyltransferase assay

Reaction mixtures containing 50 mM Tris–HCl (pH 8.0), 5 mM dithiothreitol, 50 μM AdoMet, 10 nM [^32^P]-labeled G**p**ppRNA, and TbCmt1 as specified were incubated at 27°C. Aliquots were taken at time specified and the reaction was terminated by heating the sample at 80°C for 5 min. Samples were digested with 0.5 μg of nucleotide pyrophosphatase for 60 min at 37°C, and products were separated on PEI thin-layer chromatography (TLC) plates, which were developed with 0.45 M ammonium sulfate as described (35). The extent of methylation of the cap (^m7^Gp/[^m7^Gp + Gp]) was quantitated by scanning the TLC plate with the PhosphorImager.

### Expression of TbCmt1-PTP in *T. brucei*

A Protein C-TEV-Protein A (PTP) tag was inserted into TbCmt1 loci by homologous recombination (38). The TbCmt1 region (1–942 nt) was amplified with forward primers that introduce an ApaI restriction site and reverse primer that disrupts the stop codon and introduces a NotI restriction site, and the PCR fragment was ligated into pC-PTP-Neo in frame with the PTP-tag (38). The plasmid was linearized using Nsi1 and electroplated into the *T. brucei brucei* EATRO164 cell line and was selected by G418 (15 μg/ml). Expression of TbCmt1-PTP fusion protein was confirmed by Western blot. Detection of TbCmt1 on fluorescence microscopy was performed as described (34). The PTP-fusion protein was detected using mouse THE™ Protein C Tag antibody (Genscript; 1:500 dilution), and Alexa Fluor 568 mouse IgG secondary antibody. The endogenous TbCmt1 was detected by rabbit anti-TbCmt1 antibody (19) and Alexa Fluor 488 anti-rabbit IgG secondary antibody. The cells were resuspended in 10 μl of PBS and mixed with an equal volume of mounting buffer, which contained 30 μM of 4′,6-diamidino-2-phenylindole (DAPI) and an anti-quencher that minimized the fluorescent bleaching.

### Data analysis

Radioisotope signals were detected by Fujix BAS-2500 PhosphorImager and quantitated by Multi Gauge Ver. 3.0 (Fuji Photo Film, Tokyo, Japan). Data analysis was performed using GraphPad Prism 8 (GraphPad Software, La Jolla, CA).

## Results

### TbCe1 RNA kinase activity is stimulated by hypermethylation

**Figure 1.**
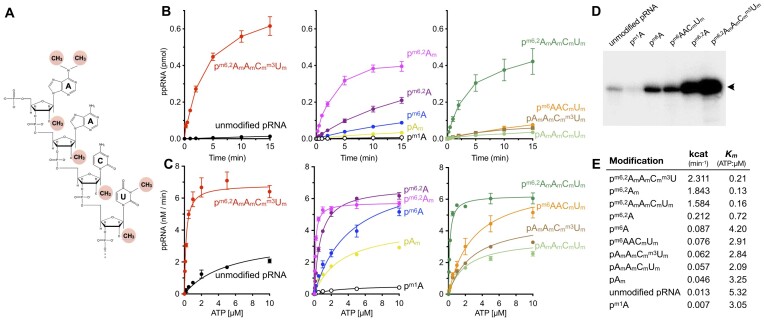
TbCe1 RNA kinase activity is enhanced by hypermethylation on the 5′ end of pRNA. (**A**) Structure of hypermethylated pRNA (p^m6,2^A_m_A_m_C_m_^m3^U_m_) derived from decapped cap 4 terminated RNA. Methylation (–CH_3_) on the first four nucleotides are highlighted in red. (**B**) Time course. Standard RNA kinase assay (100 μl) contained 50 ng of TbCe1, 1 μM of [γ-^32^P] ATP and either 100 nM of modified or unmodified pRNA as indicated. An aliquot (10 μl) was withdrawn at the indicated time. The yield of ppRNA (pmol) is plotted as a function of incubation time. The results are shown in three separate panels for clarity. *Left panel*: Effect of unmethylated RNA vs hypermethylated pRNA; *Middle panel*: Effect on pRNA modification on first nucleotide; *Right panel:* Effect on pRNA with 2′-O ribose and *N3*-uridine modifications. (**C**) ATP titration. Standard RNA kinase assay (10 μl) containing either 1 pmol of modified or unmodified pRNA was incubated with TbCe1 (20 ng) for 10 min with indicated concentrations of [γ-^32^P] ATP. The yield of the ppRNA product was plotted as a function of ATP concentration by fitting the data to non-linear regression in Prism. For clarity, ATP concentrations of up to 10 μM is shown. All experiments were performed in triplicate. Standard error bars are included for each datum point. (**D**) Standard RNA kinase assay was performed in the presence of 1 μM of [γ-^32^P] ATP and TbCe1 (5 ng) for 10 min, with 100 nM of indicated modified or unmodified pRNAs. A 18% urea-gel scanned with PhosphorImager is shown. Additional gels are shown in Supplementary Figures S2 and S3. (**E**) Turnover numbers (*k*_cat_) were determined from the initial rate of phosphorylation on pRNA substrates presented in panel (B). *V*_max_ and *K*_m_ for ATP were calculated from the experiments shown in Supplementary Figure S3.

All the mature mRNA in the trypanosome is capped and hypermethylated at the 5′-end, acquired through trans-splicing of a SL RNA. If TbCe1 acts as a recapping enzyme to convert decapped mRNA, a physiological substrate for the TbCe1 should preserve all the methylations present on the cap 4 structure with 5′-monophosphate end (Figure 1A). We previously showed that SL pRNA with 2′-*O* methylations on the first or all four nucleotides could enhance the RNA kinase activity of TbCe1 by up to 3-fold compared to the unmethylated pRNA (34). To address whether additional modification derived from cap 4 can influence the TbCe1 enzymatic activity, we chemically synthesized 21-mer pRNA oligonucleotide which represents decapped trypanosome mRNA with *N6,N6*-*2′-O* trimethylation on the first adenosine, *2′-O* methylations at the 2nd adenosine and the 3rd cytosine, and *N3-2′-O* dimethylation on the fourth uracil (p^m6,2^A_m_A_m_C_m_^m3^U_m_ RNA; Figure 1A). TbCe1 RNA kinase activity was assayed in the presence of 1 μM [**γ**-^32^P] ATP and 0.5 mM Mg^2+^, and the ^32^P-labeled ppRNAs were visualized using PAGE. The initial rate of phosphorylation on the p^m6,2^A_m_A_m_C_m_^m3^U_m_ RNA was two orders of magnitude higher compared to the unmodified pRNA (Figure 1B; Supplementary Figure S2). The *kcat* for p^m6,2^A_m_A_m_C_m_^m3^U_m_ RNA was 2.3 min^−1^, compared to 0.057 min^−1^ on pA_m_A_m_C_m_U_m_, 0.046 min^−1^ on pA_m_, and 0.013 min^−1^ on the unmodified pRNA. The stimulation was most pronounced at limited ATP concentrations. The *K*_m_ values of ATP in the reaction containing p^m6,2^A_m_A_m_C_m_^m3^U_m_, pA_m_A_m_C_m_U_m_, pA_m_, and unmodified pRNAs were 0.21, 2.09, 3.25 and 5.32 μM, respectively (Figure 1C and E). The *V*max increased to 6.8 nM/min on a fully methylated p^m6,2^A_m_A_m_C_m_^m3^U_m_ pRNA substrate, compared to 3.5 nM/min on pA_m_A_m_C_m_U_m_ and unmodified pRNA (Figure 1C; Supplementary Figure S3). These results strengthen the notion that hypermethylated pRNA derived from cap 4 mRNA, is a physiological target for recapping by TbCe1.

### Recapping activity is stimulated by *N6,N6 2*′*-O* trimethylation on the first adenosine

We synthesized a series of pRNA substrates having one or more methylations to determine which cap 4 modification is accountable for the stimulatory effect on TbCe1 (Table 1). Based on the above result, modifications on the nucleotide bases are required for the optimal RNA kinase activity. The pRNAs with *N6,N6 2*′*-O* trimethylation on the first adenosine, p^m6,2^A_m_ (1.843 min^−1^) and p^m6,2^A_m_A_m_C_m_U_m_ (1.584 min^−1^), which lacks *N3-*methylation on the fourth uracil (^m3^U), were efficiently phosphorylated by TbCe1, greater than 100-fold compared to the unmodified pRNA (Figure 1B; Supplementary Figure S2). The *Km*_ATP_ on p^m6,2^A_m_ and p^m6,2^A_m_A_m_C_m_U_m_ were less than 0.2 μM (Figure 1C and E). The kinase activity was reduced on p^m6,2^A RNA (0.212 min^−1^), which lacks the 2′-O ribose methylation on the 1st nucleotide yet maintained ∼20-fold higher activity than the unmodified pRNA. Comparison on the rate of phosphorylation on p^m6,2^A_m_A_m_C_m_^m3^U_m,_ p^m6,2^A_m_A_m_C_m_U_m,_ pA_m_A_m_C_m_^m3^U_m_ and pA_m_A_m_C_m_U_m_ RNAs suggests that ^m3^U modification moderately upregulates the activity (Figure 1B and E). These results demonstrate that modifications on the first adenosine are primarily responsible for the stimulatory effect, and 2′-*O* ribose and ^m3^U modifications further enhance the kinase activity.

To further evaluate the effect of methylations on the first nucleotide, we synthesized pRNA oligonucleotides with a following modification: *N6*-methyladenosine (p^m6^A) at position 1, *N6*-methyladenosine with 2′-*O* ribose methylations at positions 3 and 4 (p^m6^AAC_m_U_m_), and a *N1*-methyladenosine (p^m1^A) at position 1. An ^m6^A modification partially activated the enzyme (0.087 min^−1^), albeit less than ^m6,2^A modification (Figure 1B and E). Each methylation on the first adenosine additively enhances the kinase activity. Activation was specific to *N6*-methylation as *N1*-methyladenosine was inhibitory to the reaction (0.007 min^−1^, Figure 1B and D; Supplementary Figures S2 and S3). Consistent with the above findings, the kinase activity on p^m6^AAC_m_U_m_ (0.076 min^−1^) was similar on p^m6^A, which implies that 2′-O ribose modifications at position 3 and 4 do not have significant effect when m6A modification is present (Figure 1B and C).

### Effect of RNA methylations on guanylyltransferase activity

**Figure 2.**
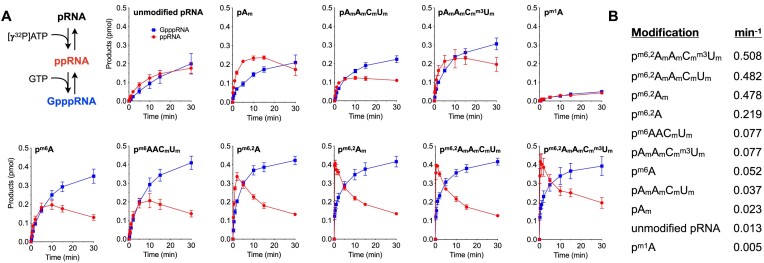
GpppRNA formation on hypermethylated pRNAs by TbCe1. (**A**) Two-step RNA recapping reaction with TbCe1. Reaction mixture (100 μl) contained 50 mM Tris–HCl pH 8.5, 1 mM dithiothreitol, 0.5 mM MgCl_2_, 500 ng of TbCe1, 10 μM [γ-^32^P] ATP, 10 μM GTP with 100 nM of pRNA with or without modifications as indicated and listed on Table 1. An aliquot (10 μl) was withdrawn at the indicated time and products were separated on PAGE. The yields of indicated ppRNA intermediate (red) and GpppRNA (blue) are plotted as a function of incubation time. (**B**) The initial rate of GpppRNA formation on modified pRNAs substrates from the data presented in (A). Representative gels are shown in Supplementary Figure S4.

To address whether hypermethylation could affect subsequent reaction to form GpppRNA, we performed a recapping assay on modified pRNAs in the presence of 10 μM GTP and 10 μM [γ-^32^P] ATP. The concentration of ATP was increased to 10 μM to evaluate the recapping activities on all the modified RNA substrates. The pRNAs were converted to ppRNA intermediates evinced by the appearance of ^32^P-labeled ppRNA species at earlier time points and declined with a concomitant increase of ^32^P-labeled GpppRNA (Figure 2A; Supplementary Figure S4). As expected, the rate of GpppRNA formation was faster on modified pRNAs which were efficiently phosphorylated to form ppRNA intermediate. The pRNAs with *N6,N6 2′-O* trimethyladenosine were enhanced by up to 40-fold compared to the unmodified pRNA. Except for p^m1^A, all other pRNA modifications enhanced the overall GpppRNA formation: p^m6,2^A (17-fold) > p^m6^AAC_m_U_m_ (6-fold) > p^m6^A (4-fold) > pA_m_A_m_C_m_U_m_ (2.9-fold) and pA_m_ (1.8-fold). The turnover numbers of GpppRNA formation on the modified pRNA substrates are summarized in Figure 2B. We noted that A_m_ modification at position 1 could be inhibitory for the step 2 guanylation, as ppA_m_ RNA intermediate accumulated in the reaction and was slowly converted to GpppA_m_RNA.

**Figure 3.**
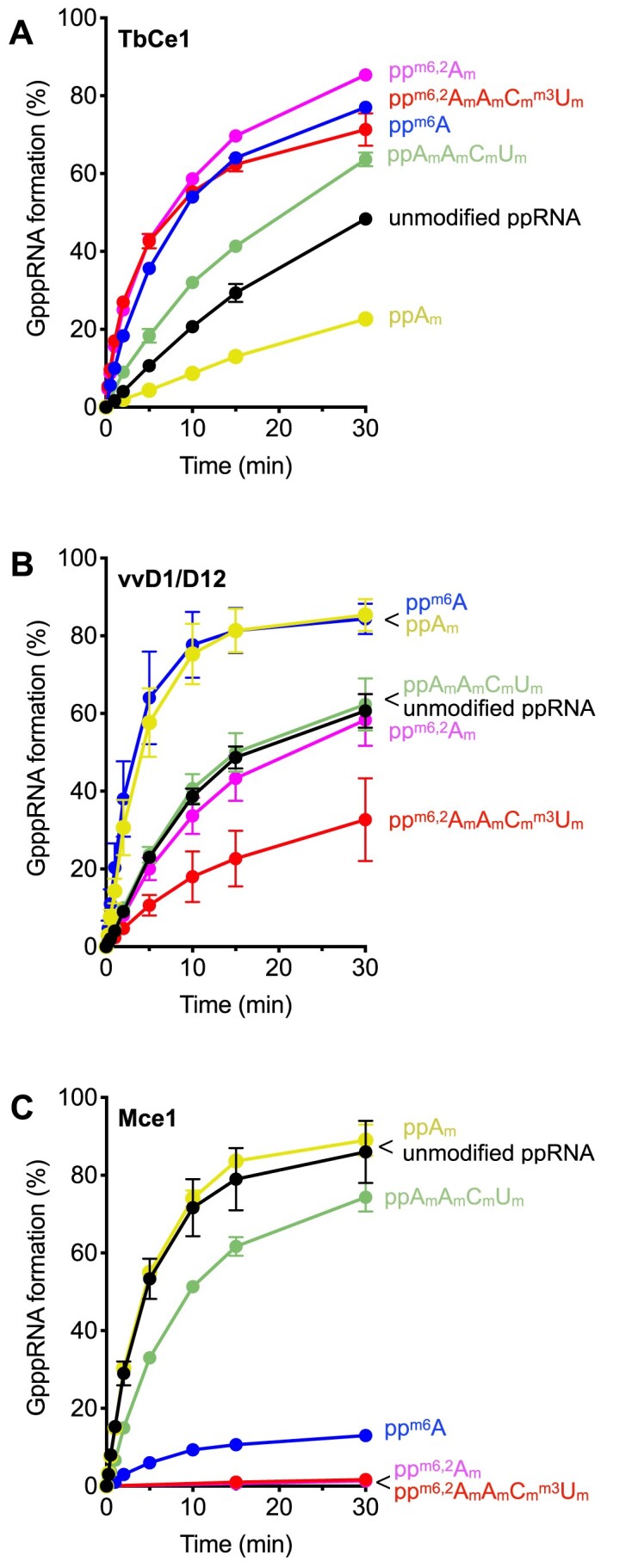
Effect of RNA methylation on the guanylyltransferase activities. Guanylyltransferase assay was performed in a reaction mixture (20 μl) containing 50 mM Tris–HCl pH 8.5, 1 mM dithiothreitol, 2.5 mM MgCl_2_, 50 μM GTP and 50 nM modified and unmodified ^32^P-labeled ppRNA as indicated, was incubated with either (**A**) TbCe1 (100 ng at 27°C), (**B**) vaccinia virus capping enzyme D1/D12 (4 units at 37°C) or (**C**) mammalian capping enzyme Mce1 (100 ng at 37°C). Aliquots (2 μl) were removed at time indicated and the reaction was terminated by addition of 1 μl of 0.5 M EDTA. Products were digested with nuclease P1 and separated on the PEI thin-layer chromatography (TLC) plates. Percent GpppRNA formed is plotted as a function of incubation time. The data shown represents the average of three separate experiments with SE bars.

To further evaluate the effect of hypermethylation on guanylyltransferase activity, we prepared a number of ^32^P-labeled diphosphate-terminated RNA substrates and assayed for a conversion of ppRNA to GpppRNA (Figure 3A). The initial rate of GpppRNA formation on the unmodified ppRNA was 0.015 min^−1^. The rate of guanylation was enhanced on pp^m6,2^A_m_A_m_C_m_^m3^U_m_ (8.2-fold increase), pp^m6,2^A_m_ (6.3-fold), and pp^m6^A (4.4-fold) (Figure 3A). Approximately 2-fold enhancement was observed on ppA_m_A_m_C_m_U_m_ RNA. We noted that ppA_m_ modification was inhibitory for the guanylyltransferase activity (2.5-fold decrease), consistent with the overall recapping assay in which ppRNA intermediate accumulates with pA_m_ RNA substrate (Figure 2A; Supplementary Figure S4). These results suggest that hypermethylation could stimulate both the RNA kinase and the guanylyltransferase activities of TbCe1. TbCe1 could discriminate recapping the undermethylated (pRNA or pA_m_ RNA) versus the hypermethylated RNA.

The guanylyltransferase domain of TbCe1 is homologous with other capping enzymes, including vaccinia virus capping enzyme (vvD1/D12) and mammalian capping enzyme (Mce1/*RNGTT*) (19,39–41). All three guanylyltransferases share mechanistic similarities in transferring of GMP to ppRNA via formation of enzyme-GMP complex. We therefore addressed whether hypermethylation at the 5′-end of the ppRNA could affect the vvD1/12 and Mce1 activities (Figure 3B and C). The vvD1/D12 activity was enhanced by ^m6^A and A_m_ modification on the first nucleotide, while ppA_m_A_m_C_m_U_m_ and pp^m6,2^A_m_ modifications did not have significant impact (Figure 3B). The activity was reduced by ∼ 2-fold on a fully methylated pp^m6,2^A_m_A_m_C_m_^m3^U_m_, implying that hypermethylation does not significantly affect the vaccinia enzyme. In contrast, Mce1 was completely inert for transferring GMP to pp^m6,2^A_m_A_m_C_m_^m3^U_m_ and pp^m6,2^A_m_ RNAs, and was severely inhibited by the presence of ^m6^A methylation at position 1 (Figure 3C), while A_m_ methylation did not have major impact on Mce1 activity. Mammalian and vaccinia mRNAs are known to possess ^m6^A and A_m_ modifications adjacent to the cap structure. While A_m_ modification on the first nucleotide could interfere with the guanylation of ppRNA by TbCe1, it does not have significant effect with vvD1/12 and Mce1. In contrast, ^m6^A methylation on the first nucleotide enhanced guanylation by TbCe1 and vvD1/12, but the same modification was inhibitory for Mce1. We conclude that the presence of methylations at the 5′ end of RNA could influence the guanylyltransferase activity, but the impact of these methylations varies among different species. The result also suggested that recapping activities could be regulated by RNA methylation in mammalian cells, as Mce1 could act as both an mRNA cap forming enzyme in nucleus as well as recapping enzyme in the cytoplasm (32,42).

### TbCmt1 is a cytoplasmic cap methyltransferase that preferentially acts on hypermethylated GpppRNA

**Figure 4.**
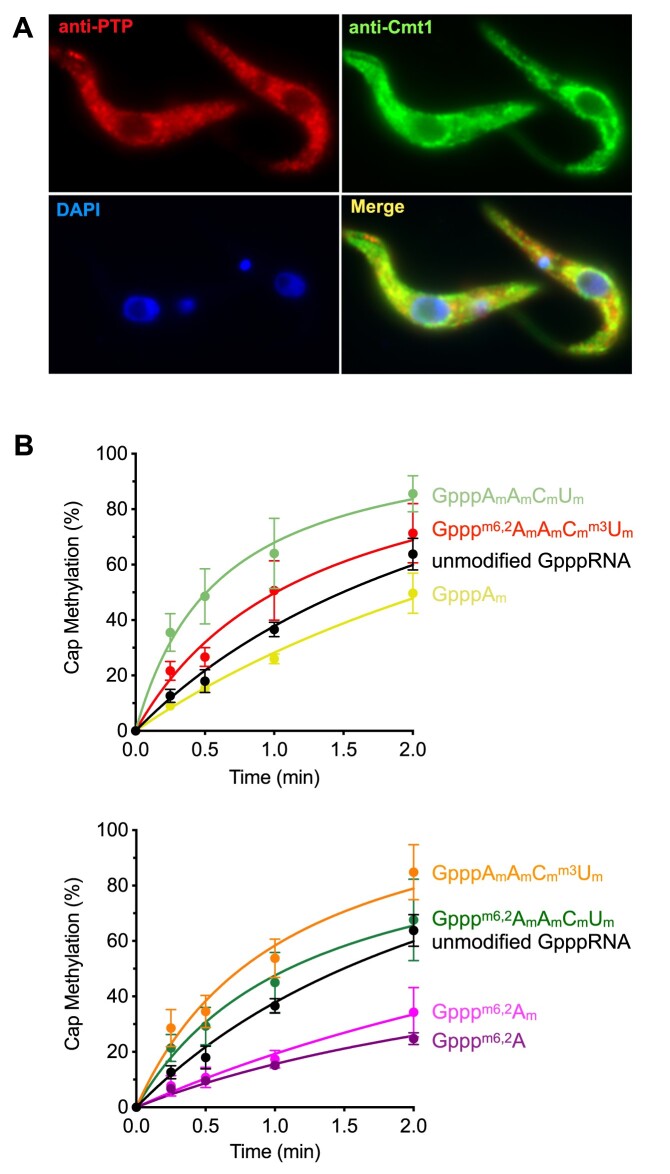
TbCmt1 is localized in the cytoplasm and the ^m7^G cap methyltransferase activity is enhanced by 2′-*O* ribose methylations. (**A**) Localization of TbCmt1. Procyclic trypanosomes expressing TbCmt1-PTP were fixed and permeabilized, and the PTP-tagged TbCmt1 and endogenous TbCmt1 were detected using immunofluorescence. The kinetoplast and nuclear DNA were counterstained with DAPI. A typical image is shown. (**B**) Effect of hypermethylation on TbCmt1 activity. Cap methyltransferase assay was performed in reaction mixture (30 μl) containing 0.5 pmol of indicated ^32^P-labeled GpppRNA and 4.5 ng of TbCmt1. Aliquots (3μl) were withdrawn at time indicated, digested with nucleotide pyrophosphatase and analyzed with TLC. Cap methylation (%) was plotted as a function of time. The data shown represents the average of three separate experiments with standard error bars.

To regenerate mature capped RNA from a decapped mRNA, the GpppRNA formed by TbCe1 must be methylated at the cap N7-guanine to form ^m7^GpppRNA. Trypanosomes encode two separate ^m7^G cap RNA methyltransferases, TbCgm1 and TbCmt1 (19,20,35). TbCgm1 was localized in the nucleus and was responsible for ^m7^G cap formation on the SL RNA (19,20,34). To address if TbCmt1 acts as a recapping enzyme, we examined the localization of TbCmt1 using indirect immunofluorescence microscopy. We generated a procyclic cell line in which TbCmt1 was C-terminally tagged with PTP epitope-tag (Supplementary Figure S5). We also detected endogenous TbCmt1 protein using an antibody raised against TbCmt1 protein. In both cases, fluorescent signals were scattered in the cytoplasm, suggesting that TbCmt1 may function in recapping (Figure 4A).

Having established that TbCe1 activities are enhanced by methylations at the 5′-end of RNA, we prepared series of modified ^32^P-GpppRNAs and assayed for a conversion to ^m7^GpppRNA by TbCmt1 (Figure 4B). TbCmt1 N7-guanine cap methyltransferase activity was most active on GpppRNA with 2′-O ribose methylations on all four nucleotides. The rate of *N7*-guanine cap methylation on the optimal GpppA_m_A_m_C_m_U_m_ RNA was 160 fmol/min/ng of TbCmt1, which corresponds to a turnover number of 5.9 min^−1^ and was 3.7-fold faster than the unmodified GpppRNA (1.7 min^−1^). The optimal GpppRNA substrates for TbCmt1 were: GpppA_m_A_m_C_m_U_m_ (100%) > GpppA_m_A_m_C_m_^m3^U_m_ (80%) > Gppp^m6,2^A_m_A_m_C_m_U_m_ (60%) > Gppp^m6,2^A_m_A_m_C_m_^m3^U_m_ (42%) > unmodified GpppRNA (27%) > GpppA_m_ (19%) > Gppp^m6,2^A_m_ (14%) ≥ Gppp^m6,2^A (13%) RNA. These results suggest that presence of 2′-*O* ribose methylations on the four nucleotides enhances the TbCmt1 activity; however, modification on the nucleotide bases could reduce the activity.

**Figure 5.**
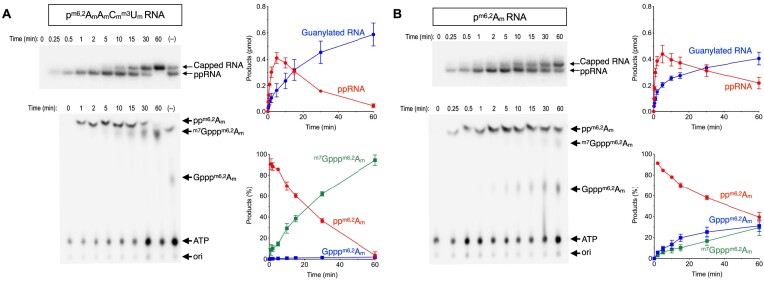
Kinetic analysis of recapping hypermethylated pRNA by TbCe1 and TbCmt1. (**A**) Cap formation on the hypermethylated p^m6,2^A_m_A_m_C_m_^m3^U_m_ RNA substrate. Reaction mixture (100 μl) containing 50 mM Tris–HCl (pH 8.5), 1 mM dithiothreitol, 0.5 mM MgCl_2_, 10 μM [γ-^32^P] ATP, 50 μM AdoMet, 100 nM of p^m6,2^A_m_A_m_C_m_^m3^U_m_ RNA, TbCe1 (50 ng) and TbCmt1 (20 ng) was incubated at 27°C. Aliquots were withdrawn at each time point and analyzed by 18% Urea-PAGE (top panel) and TLC (bottom panel). A control reaction lacking TbCmt1 at 60 min time point is indicated on the right lane (–). Positions of ^32^P-labeled capped RNA and ppRNA intermediate are indicated. The yields of ppRNA intermediate and guanylated RNA (^m7^GpppRNA and GpppRNA) were plotted as a function of time (shown to the right of representative gel). For TLC analysis, products were extracted with phenol-chloroform and precipitated with ethanol. Samples were then digested by nuclease P1 and separated on TLC plate, which was developed in 1 M formic acid and 0.5 M LiCl. The positions of pp^m6,2^A_m_, Gppp^m6,2^A_m_, ^m7^Gppp^m6,2^A_m_, and ATP are indicated. The products (%) were plotted as a function of time (shown to the right of representative TLC plate). The data shown on the graph represents the average of three separate experiments with SE bars. (**B**) Cap formation on the partially methylated p^m6,2^A_m_ RNA. Identical to (A) except that p^m6,2^A_m_ RNA was used as a substrate.

To further evaluate the effect of 2′-*O* ribose methylations on TbCmt1, we performed a kinetic analysis of ^m7^G cap formation by incubating both TbCe1 and TbCmt1 together with either hypermethylated p^m6,2^A_m_A_m_C_m_^m3^U_m_ or undermethylated p^m6,2^A_m_ RNA. These RNA substrates were selected because they can be efficiently phosphorylated and guanylated to form Gppp^m6,2^A_m_A_m_C_m_^m3^U_m_ and Gppp^m6,2^A_m_ RNA (Figure 2). On the p^m6,2^A_m_A_m_C_m_^m3^U_m_ RNA, all the input substrate was guanylated by 60 min (Figure 5A, top panels). Analysis of 5′-end by nuclease P1 digestion reveals that majority of the guanylated RNAs were methylated to ^m7^Gppp^m6,2^A_m_, suggesting that guanine N7 position is methylated as soon as Gppp^m6,2^A_m_A_m_C_m_^m3^U_m_ RNA is formed (Figure 5A, bottom panels). In contrast, guanylation of p^m6,2^A_m_ RNA reached a plateau after 10 min (Figure 5B, top panels), and less than half of the guanylated RNA was methylated at the N7 position to form ^m7^Gppp^m6,2^A_m_ (Figure 5B, bottom panels). The rate of ^m7^GpppRNA formation on p^m6,2^A_m_A_m_C_m_^m3^U_m_ was ∼ 3-fold faster than p^m6,2^A_m_ RNA, consistent with the results presented in Figure 4B, that 2′-*O* ribose methylations function to activate TbCmt1 cap methylation.

To address whether hypermethylation on 2′-*O* ribose could enhance the cap methylation by other cap methyltransferase, we repeated the above assay using the vaccinia virus capping enzyme, vvD1/D12. Because vvD1/D12 is not capable of phosphorylating pRNA and the guanylyltransferase activity was inhibited on pp^m6,2^A_m_A_m_C_m_^m3^U_m_ modifications (Figure 3B), the cap methyltransferase activity was compared on ppA_m_A_m_C_m_U_m_ and unmodified ppRNAs (Supplementary Figure S6). The 2′-*O* ribose modifications did not enhance vvD1/D12 cap methyltransferase activity, rather, it was slightly inhibitory as it accumulated GpppA_m_A_m_C_m_U_m_ RNA intermediates in the reaction. Thus, the enhanced guanine *N7* methyltransferase activity on GpppA_m_A_m_C_m_U_m_ RNA was specific to TbCmt1.

In summary, we conclude that trypanosome cytoplasmic capping enzymes recognize distinct methyl groups within the cap 4 structure to promote mRNA recapping. Modifications on the first adenine, ^m6,2^A_m_, activate the RNA kinase and the guanylyltransferase of TbCe1 to convert decapped pRNA to GpppRNA. In contrast, hypermethylation on 2′-*O* ribose activates guanine *N7* methyltransferase activity of TbCmt1 to convert GpppRNA to ^m7^GpppRNA, to regenerate a mature cap structure on decapped mRNA.

## Discussion

Our results suggest a distinct role of cap 4 modifications in recruitment and activation of mRNA recapping enzymes in trypanosomes. The methyl groups derived from cap 4 could function to stimulate the catalytic activity of TbCe1 and TbCmt1 recapping enzymes. Specifically, we found that TbCe1 RNA kinase activity is enhanced by 2 orders of magnitude by ^m6,2^A_m_ trimethylation on the first nucleotide of pRNA. Each methyl group on the first adenosine can synergistically enhance the RNA kinase activity. The guanylyltransferase activity is also enhanced by hypermethylation. However, when ribose methylation is the sole modification on the first adenosine, it negatively impacts the activity. The uncapped pRNAs with ^m6^A modification had higher *V*max compared to the pRNA lacking ^m6^A on the first adenosine. We speculate that the rate of reversible reactions could be faster on the pRNA that lacks ^m6^A modification.

Most eukaryotic organisms encode multiple decapping enzymes with different specificity (30). Trypanosomes have two decapping enzymes: a Nudix hydrolase that releases ^m7^Gpp that leaves pRNA (34), and an ApaH-like phosphatase that releases ^m7^Gp to form ppRNA (43). The hypermethylated ppRNA can also enhance the guanylyltransferase activity of TbCe1, which implies that decapped ppRNA generated by ApaH-like phosphatase can be recapped by TbCe1. Our findings suggest that recapped mRNA will preserve the hypermethylation at the 5′-end. Because cap 4 modification precedes SL RNA capping and trans-splicing in the nucleus, the removal of ^m7^Gpp or ^m7^Gp by decapping enzyme will preserve the hypermethylation originating from cap 4. We found that mRNA recapping activities are stimulated by hypermethylation, which ensures that recapping only takes place on mRNA that has undergone decapping and not with any other RNAs with 5′-monophosphate or diphosphate ends.

To regenerate a mature mRNA, the GpppRNA generated by TbCe1 needs to be irreversibly methylated at guanine *N7* position by cap methyltransferase. We showed that TbCmt1 is localized in the cytoplasm and its cap guanine *N7* methyltransferase activity was enhanced by 2′-*O* ribose methylations derived from cap 4. While TbCmt1 is not an essential gene in cultured procyclic (19) or blood form of parasites (44), mice infected with TbCmt1 null mutant showed reduced parasite proliferation suggesting that TbCmt1 gene is required to establish parasitemia (44). We hypothesize that TbCe1 and TbCmt1 act together as a recapping enzyme for the expression of specific mRNA in response to stress or sudden environmental changes. Indeed, TbCmt1 is found in the stress granules (45), a cytoplasmic compartment that functions to regulate the availability of mRNA in response to stress or sudden enviromental changes, including transmission between hosts and exposure to the host immune system. Further investigation is needed to determine what kind of transcripts are being targeted for recapping and how cap 4 methylations are regulated in the parasite.

RNA modifications are dynamically regulated to mediate diverse effects on mRNA metabolism by affecting interactions with RNA binding proteins or altering the RNA structure to change the accessibility of binding sites for regulatory factors. While the majority of SL RNA appears to be hypermethylated in the form of cap 4, a 5′-end labeling analysis of *T. brucei* mRNA suggests that significant proportion lacks 2′-*O* ribose methylation on the first adenosine (2). In mammalian cells, the ^m6^A modification adjacent to cap structure is methylated by PCIF1 (5–7) and can be demethylated by FTO (9), raising the possibility that cap 4 methylation could be reversibly regulated, although homolog of FTO has not yet been identified in trypanosomes.

The present study raises the interesting prospect that modification at the 5′-terminus could influence mRNA recapping in other eukaryotes. Mce1/RNGTT is a mammalian capping enzyme responsible for cap 0 formation in the nucleus; however, it can form a complex in the cytoplasm with Nck1 protein and a yet unidentified RNA kinase to function as a recapping enzyme (42,46). We showed that Mce1/RNGTT can cap the transcript with A_m_ on the first nucleotide, but its activity is inhibited when the transcript is modified with ^m6^A. We predict that while ^m6^A_m_ modification could protect mRNA from decapping (9), transcripts with the A_m_ modification could be more efficiently recapped in mammals. This may explain why the effect of PCIF1 knockout on mRNA stability and translation varies among experiments (5–8). PCIF1 may also act post-transcriptionally to modify uncapped mRNAs, although the efficiency is lower compared to the capped RNA (13,47). We speculate that PCIF1 and FTO could alter the cap methylation status to regulate which transcripts are preferentially decapped, recapped, or left uncapped to maintain cap homeostasis.

## Supplementary Material

gkae614_Supplemental_File

## Data Availability

All data needed to evaluate the conclusions in the paper are present in the paper and/or the Supplementary data.

## Notes

Present address: Anilkumar R. Kore, Convergence Bio Inc., 600/700 One Kendal Square, Suite 6-402 Cambridge, MA 02139, USA.

## References

[1] Perry R.P. , Kelley D.E. Existence of methylated messenger RNA in mouse L cells. Cell. 1974; 1:37–42.

[2] Dix T.C. , Haussmann I.U., Brivio S., Nallasivan M.P., HadzHiev Y., Müller F., Müller B., Pettitt J., Soller M. CMTr mediated 2′-O-ribose methylation status of cap-adjacent nucleotides across animals. RNA. 2022; 28:1377–1390. 35970556 10.1261/rna.079317.122 PMC9479742

[3] Keith J.M. , Muthukrishnan S., Moss B. Effect of methylation of the N6 position of the penultimate adenosine of capped mRNA on ribosome binding. J. Biol. Chem. 1978; 253:5039–5041. 670177

[4] Wei C.-M. , Gershowitz A., Moss B. N6, O2′-dimethyladenosine a novel methylated ribonucleoside next to the 5′ terminal of animal cell and virus mRNAs. Nature. 1975; 257:251–253. 1161029 10.1038/257251a0

[5] Akichika S. , Hirano S., Shichino Y., Suzuki T., Nishimasu H., Ishitani R., Sugita A., Hirose Y., Iwasaki S., Nureki O. et al. Cap-specific terminal N 6 -methylation of RNA by an RNA polymerase II–associated methyltransferase. Science. 2019; 363:eaav0080. 30467178 10.1126/science.aav0080

[6] Sendinc E. , Valle-Garcia D., Dhall A., Chen H., Henriques T., Navarrete-Perea J., Sheng W., Gygi S.P., Adelman K., Shi Y. PCIF1 Catalyzes m6Am mRNA methylation to regulate gene expression. Mol. Cell. 2019; 75:620–630. 31279659 10.1016/j.molcel.2019.05.030 PMC6688901

[7] Pandey R.R. , Delfino E., Homolka D., Roithova A., Chen K.-M., Li L., Franco G., Vågbø C.B., Taillebourg E., Fauvarque M.-O. et al. The mammalian cap-specific m6Am RNA methyltransferase PCIF1 regulates transcript levels in mouse tissues. Cell Rep. 2020; 32:108038. 32814042 10.1016/j.celrep.2020.108038

[8] Boulias K. , Toczydłowska-Socha D., Hawley B.R., Liberman N., Takashima K., Zaccara S., Guez T., Vasseur J.-J., Debart F., Aravind L. et al. Identification of the m6Am methyltransferase PCIF1 reveals the location and functions of m6Am in the transcriptome. Mol. Cell. 2019; 75:631–643. 31279658 10.1016/j.molcel.2019.06.006 PMC6703822

[9] Mauer J. , Luo X., Blanjoie A., Jiao X., Grozhik A.V., Patil D.P., Linder B., Pickering B.F., Vasseur J.-J., Chen Q. et al. Reversible methylation of m(6)Am in the 5’ cap controls mRNA stability. Nature. 2017; 541:371–375. 28002401 10.1038/nature21022 PMC5513158

[10] Despic V. , Jaffrey S.R. mRNA ageing shapes the Cap2 methylome in mammalian mRNA. Nature. 2023; 614:358–366. 36725932 10.1038/s41586-022-05668-z PMC9891201

[11] Markiewicz L. , Drazkowska K., Sikorski P.J. Tricks and threats of RNA viruses – towards understanding the fate of viral RNA. RNA Biol. 2021; 18:669–687. 33618611 10.1080/15476286.2021.1875680 PMC8078519

[12] Leung D.W. , Amarasinghe G.K. Structural insights into RNA recognition and activation of RIG-I-like receptors. Curr. Opin. Struct. Biol. 2012; 22:297–303. 22560447 10.1016/j.sbi.2012.03.011 PMC3383332

[13] Tartell M.A. , Boulias K., Hoffmann G.B., Bloyet L.-M., Greer E.L., Whelan S.P.J. Methylation of viral mRNA cap structures by PCIF1 attenuates the antiviral activity of interferon-β. Proc. Natl. Acad. Sci. U.S.A. 2021; 118:e2025769118. 34266951 10.1073/pnas.2025769118 PMC8307471

[14] Bangs J.D. , Crain P.F., Hashizume T., McCloskey J.A., Boothroyd J.C. Mass spectrometry of mRNA cap 4 from trypanosomatids reveals two novel nucleosides. J. Biol. Chem. 1992; 267:9805–9815. 1349605

[15] Freistadt M.S. , Cross G.A., Branch A.D., Robertson H.D. Direct analysis of the mini-exon donor RNA of Trypanosoma brucei: detection of a novel cap structure also present in messenger RNA. Nucleic Acids Res. 1987; 15:9861–9879. 2447560 10.1093/nar/15.23.9861 PMC306536

[16] Perry K.L. , Watkins K.P., Agabian N. Trypanosome mRNAs have unusual “cap 4” structures acquired by addition of a spliced leader. Proc. Natl. Acad. Sci. U.S.A. 1987; 84:8190–8194. 3120186 10.1073/pnas.84.23.8190 PMC299507

[17] Ullu E. , Tschudi C. Trans splicing in trypanosomes requires methylation of the 5’ end of the spliced leader RNA. Proc. Natl. Acad. Sci. U.S.A. 1991; 88:10074–10078. 1719544 10.1073/pnas.88.22.10074 PMC52870

[18] McNally K.P. , Agabian N. Trypanosoma brucei spliced-leader RNA methylations are required for trans splicing In Vivo. Mol. Cell. Biol. 1992; 12:4844–4851. 1406666 10.1128/mcb.12.11.4844-4851.1992 PMC360417

[19] Takagi Y. , Sindkar S., Ekonomidis D., Hall M.P., Ho C.K. Trypanosoma brucei encodes a bifunctional capping enzyme essential for cap 4 formation on the spliced leader RNA. J. Biol. Chem. 2007; 282:15995–16005. 17416901 10.1074/jbc.M701569200

[20] Ruan J.-P. , Shen S., Ullu E., Tschudi C. Evidence for a capping enzyme with specificity for the trypanosome spliced leader RNA. Mol. Biochem. Parasitol. 2007; 156:246–254. 17949828 10.1016/j.molbiopara.2007.09.001 PMC2706020

[21] Zamudio J.R. , Mittra B., Foldynová-Trantírková S., Zeiner G.M., Lukes J., Bujnicki J.M., Sturm N.R., Campbell D.A. The 2’-O-ribose methyltransferase for cap 1 of spliced leader RNA and U1 small nuclear RNA in Trypanosoma brucei. Mol. Cell. Biol. 2007; 27:6084–6092. 17606627 10.1128/MCB.00647-07 PMC1952150

[22] Arhin G.K. , Ullu E., Tschudi C. 2’-O-methylation of position 2 of the trypanosome spliced leader cap 4 is mediated by a 48 kDa protein related to vaccinia virus VP39. Mol. Biochem. Parasitol. 2006; 147:137–139. 16516986 10.1016/j.molbiopara.2006.01.011

[23] Hall M.P. , Ho C.K. Functional characterization of a 48 kDa Trypanosoma brucei cap 2 RNA methyltransferase. Nucleic Acids Res. 2006; 34:5594–5602. 17028101 10.1093/nar/gkl573 PMC1636459

[24] Arhin G.K. , Li H., Ullu E., Tschudi C. A protein related to the vaccinia virus cap-specific methyltransferase VP39 is involved in cap 4 modification in Trypanosoma brucei. RNA. 2006; 12:53–62. 16301606 10.1261/rna.2223406 PMC1370885

[25] Zamudio J.R. , Mittra B., Zeiner G.M., Feder M., Bujnicki J.M., Sturm N.R., Campbell D.A. Complete cap 4 formation is not required for viability in Trypanosoma brucei. Euk. Cell. 2006; 5:905–915. 10.1128/EC.00080-06 PMC1489268 16757738

[26] Zamudio J.R. , Mittra B., Campbell D.A., Sturm N.R. Hypermethylated cap 4 maximizes Trypanosoma brucei translation. Mol. Microbiol. 2009; 72:1100–1110. 19504740 10.1111/j.1365-2958.2009.06696.x PMC2859698

[27] Smith P. , Ho C.K., Takagi Y., Djaballah H., Shuman S. Nanomolar inhibitors of trypanosoma brucei RNA triphosphatase. mBio. 2016; 7:e00058-16. 26908574 10.1128/mBio.00058-16 PMC4791841

[28] Takagi Y. , Kuwabara N., Dang T.T., Furukawa K., Ho C.K. Crystal structures of the RNA triphosphatase from Trypanosoma cruzi provide insights into how it recognizes the 5’-end of the RNA substrate. J. Biol. Chem. 2020; 295:9076–9086. 32381506 10.1074/jbc.RA119.011811 PMC7335777

[29] Ho C.K. , Shuman S. Trypanosoma brucei RNA triphosphatase. Antiprotozoal drug target and guide to eukaryotic phylogeny. J. Biol. Chem. 2001; 276:46182–46186. 11553645 10.1074/jbc.M108706200

[30] Grudzien-Nogalska E. , Kiledjian M. New insights into decapping enzymes and selective mRNA decay. Wiley Interdiscip. Rev.: RNA. 2016; 8:e1379. 10.1002/wrna.1379 PMC5179306 27425147

[31] Coller J. , Parker R. Eukaryotic mRNA decapping. Annu. Rev. Biochem. 2004; 73:861–890. 15189161 10.1146/annurev.biochem.73.011303.074032

[32] Mukherjee C. , Patil D.P., Kennedy B.A., Bakthavachalu B., Bundschuh R., Schoenberg D.R. Identification of cytoplasmic capping targets reveals a role for cap homeostasis in translation and mRNA stability. Cell Rep. 2012; 2:674–684. 22921400 10.1016/j.celrep.2012.07.011 PMC3462258

[33] Trotman J.B. , Schoenberg D.R. A recap of RNA recapping. Wiley Interdiscip. Rev.: RNA. 2019; 10:e1504. 30252202 10.1002/wrna.1504 PMC6294674

[34] Ignatochkina A.V. , Takagi Y., Liu Y., Nagata K., Ho C.K. The messenger RNA decapping and recapping pathway in Trypanosoma. Proc. Natl. Acad. Sci. U.S.A. 2015; 112:6967–6972. 26038549 10.1073/pnas.1424909112 PMC4460440

[35] Hall M.P. , Ho C.K. Characterization of a Trypanosoma brucei RNA cap (guanine N-7) methyltransferase. RNA. 2006; 12:488–497. 16431985 10.1261/rna.2250606 PMC1383586

[36] Ho C.K. , Sriskanda V., McCracken S., Bentley D., Schwer B., Shuman S. The guanylyltransferase domain of mammalian mRNA capping enzyme binds to the phosphorylated carboxyl-terminal domain of RNA polymerase II. J. Biol. Chem. 1998; 273:9577–9585. 9545288 10.1074/jbc.273.16.9577

[37] Leiter J. , Reichert D., Rentmeister A., Micura R. Practical synthesis of cap-4 RNA. ChemBioChem. 2020; 21:265–271. 31626389 10.1002/cbic.201900590 PMC7004131

[38] Schimanski B. , Nguyen T.N., Günzl A. Highly efficient tandem affinity purification of trypanosome protein complexes based on a novel epitope combination. Euk. Cell. 2005; 4:1942–1950. 10.1128/EC.4.11.1942-1950.2005 PMC1287860 16278461

[39] Kyrieleis O.J.P. , Chang J., Peña M.D.l., Shuman S., Cusack S. Crystal structure of vaccinia virus mRNA capping enzyme provides insights into the mechanism and evolution of the capping apparatus. Structure. 2014; 22:452–465. 24607143 10.1016/j.str.2013.12.014 PMC4010090

[40] Chu C. , Das K., Tyminski J.R., Bauman J.D., Guan R., Qiu W., Montelione G.T., Arnold E., Shatkin A.J. Structure of the guanylyltransferase domain of human mRNA capping enzyme. Proc. Natl. Acad. Sci. U.S.A. 2011; 108:10104–10108. 21636784 10.1073/pnas.1106610108 PMC3121809

[41] Silva E. , Ullu E., Kobayashi R., Tschudi C. Trypanosome capping enzymes display a novel two-domain structure. Mol. Cell. Biol. 1998; 18:4612–4619. 9671471 10.1128/mcb.18.8.4612 PMC109047

[42] Otsuka Y. , Kedersha N.L., Schoenberg D.R. Identification of a cytoplasmic complex that adds a cap onto 5’-monophosphate RNA. Mol. Cell. Biol. 2009; 29:2155–2167. 19223470 10.1128/MCB.01325-08 PMC2663312

[43] Kramer S. The ApaH-like phosphatase TbALPH1 is the major mRNA decapping enzyme of trypanosomes. PLoS Pathog. 2017; 13:e1006456. 28628654 10.1371/journal.ppat.1006456 PMC5491325

[44] Kelner A. , Tinti M., Guther M.L.S., Foth B.J., Chappell L., Berriman M., Cowling V.H., Ferguson M.A.J. The mRNA cap methyltransferase gene TbCMT1 is not essential in vitro but is a virulence factor in vivo for bloodstream form Trypanosoma brucei. PLoS One. 2018; 13:e0201263. 30040830 10.1371/journal.pone.0201263 PMC6057678

[45] Fritz M. , Vanselow J., Sauer N., Lamer S., Goos C., Siegel T.N., Subota I., Schlosser A., Carrington M., Kramer S. Novel insights into RNP granules by employing the trypanosome's microtubule skeleton as a molecular sieve. Nucleic Acids Res. 2015; 43:8013–8032. 26187993 10.1093/nar/gkv731 PMC4652759

[46] Mukherjee C. , Bakthavachalu B., Schoenberg D.R. The cytoplasmic capping complex assembles on adapter protein nck1 bound to the proline-rich C-terminus of Mammalian capping enzyme. PLoS Biol. 2014; 12:e1001933. 25137142 10.1371/journal.pbio.1001933 PMC4138027

[47] Yu D. , Dai N., Wolf E.J., Corrêa I.R., Zhou J., Wu T., Blumenthal R.M., Zhang X., Cheng X. Enzymatic characterization of mRNA cap adenosine-N6 methyltransferase PCIF1 activity on uncapped RNAs. J. Biol. Chem. 2022; 298:101751. 35189146 10.1016/j.jbc.2022.101751 PMC8931429

