# Supplementary material for: Trypanosome mRNA recapping is triggered by hypermethylation originating from cap 4
Source: Nucleic Acids Res. 2024 Jul 16;52(17):10645–53. doi: 10.1093/nar/gkae614 (PMC11417388; doi:10.1093/nar/gkae614)
Supplement: gkae614_Supplemental_File [file gkae614_supplemental_file.pdf]

Supplementary Data

Trypanosome mRNA recapping is triggered by hypermethylation originating from cap 4

Anna V. Ignatochkina, Jesavel A. Iguchi, Anilkumar R. Kore, and C. Kiong Ho*

* To whom correspondence should be addressed. Tel: +81-29-853-5612; Email: kiongho@md.tsukuba.ac.jp

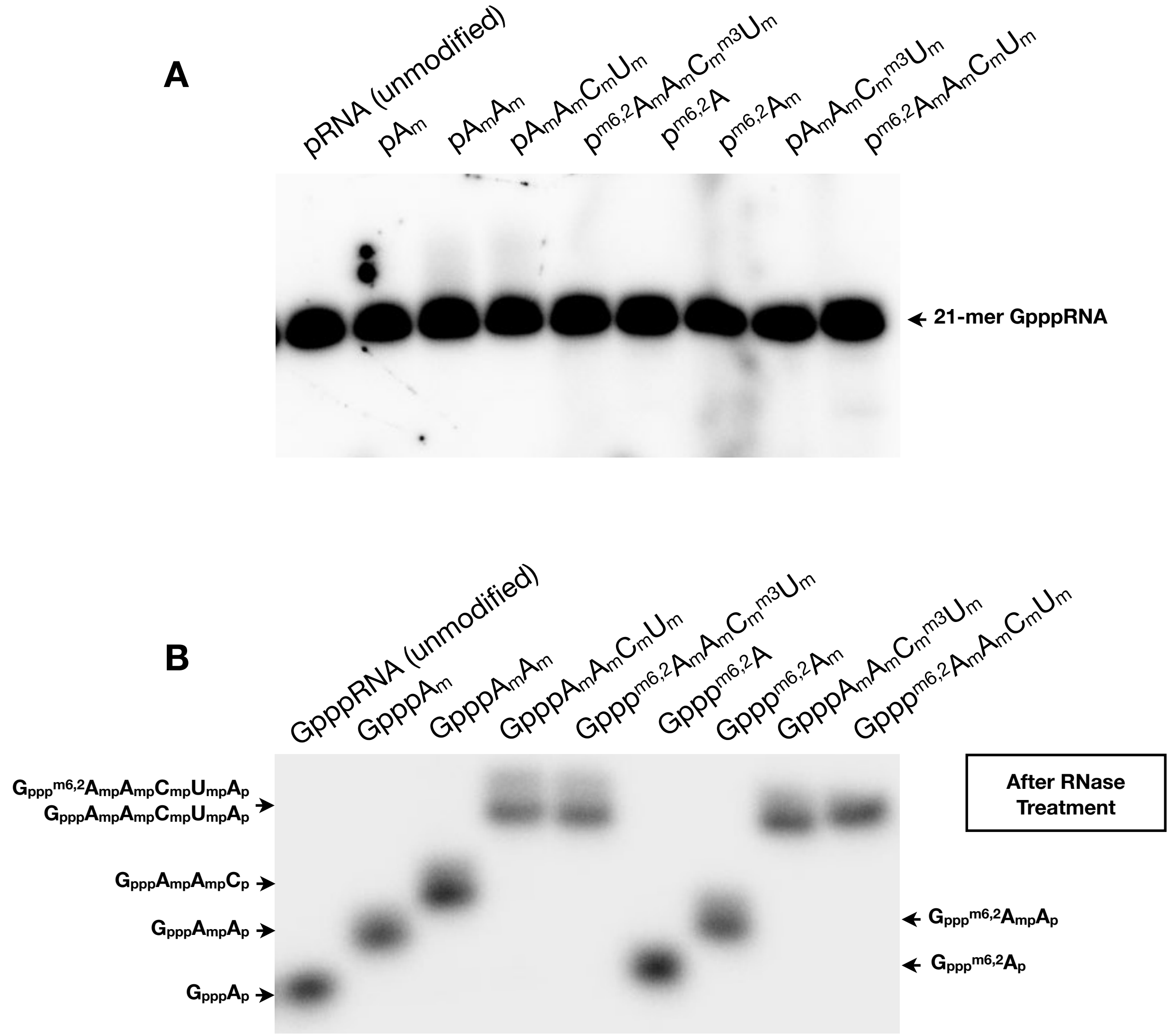

Supplementary Figure S1. Analysis of 2'-O ribose methylation on the RNA substrates. Unmodified and modified 21-mer pRNAs (pA_m, pA_mA_m, $pA_mA_mC_mU_m$, $p^{m6,2}A_mA_mC_m{}^{m3}U_m$, $p^{m6,2}A$, $p^{m6,2}A_m$, $pA_mA_mC_m{}^{m3}U_m$, $p^{m6,2}A_mA_mC_mU_m$) were 5'-capped labeled by TbCe1 (Materials and Methods). Control RNAs with 2'-*O* ribose methylations on the first two adenines (pA_mA_m) and $p^{m6,2}A$ were included to establish the position of $G_{ppp}A_{mp}A_{mp}$ and $G_{ppp}{}^{m6,2}A_p$ respectively. The $p^{m6}AAC_mU_m$ was excluded because this analysis cannot determine ribose methylation at 3rd and 4th positions. (A) Aliquots of purified G**p**ppRNAs were separated on 18% PAGE. The PhosphorImager image of the gel is shown. (B) Aliquots of purified G**p**ppRNAs indicated were digested with an RNase cocktail (mixture of RNase A, RNase T1 and RNase T2), and products were resolved by denaturing 21% PAGE. The PhosphorImager image of the gel is shown. Positions of digested products are indicated.

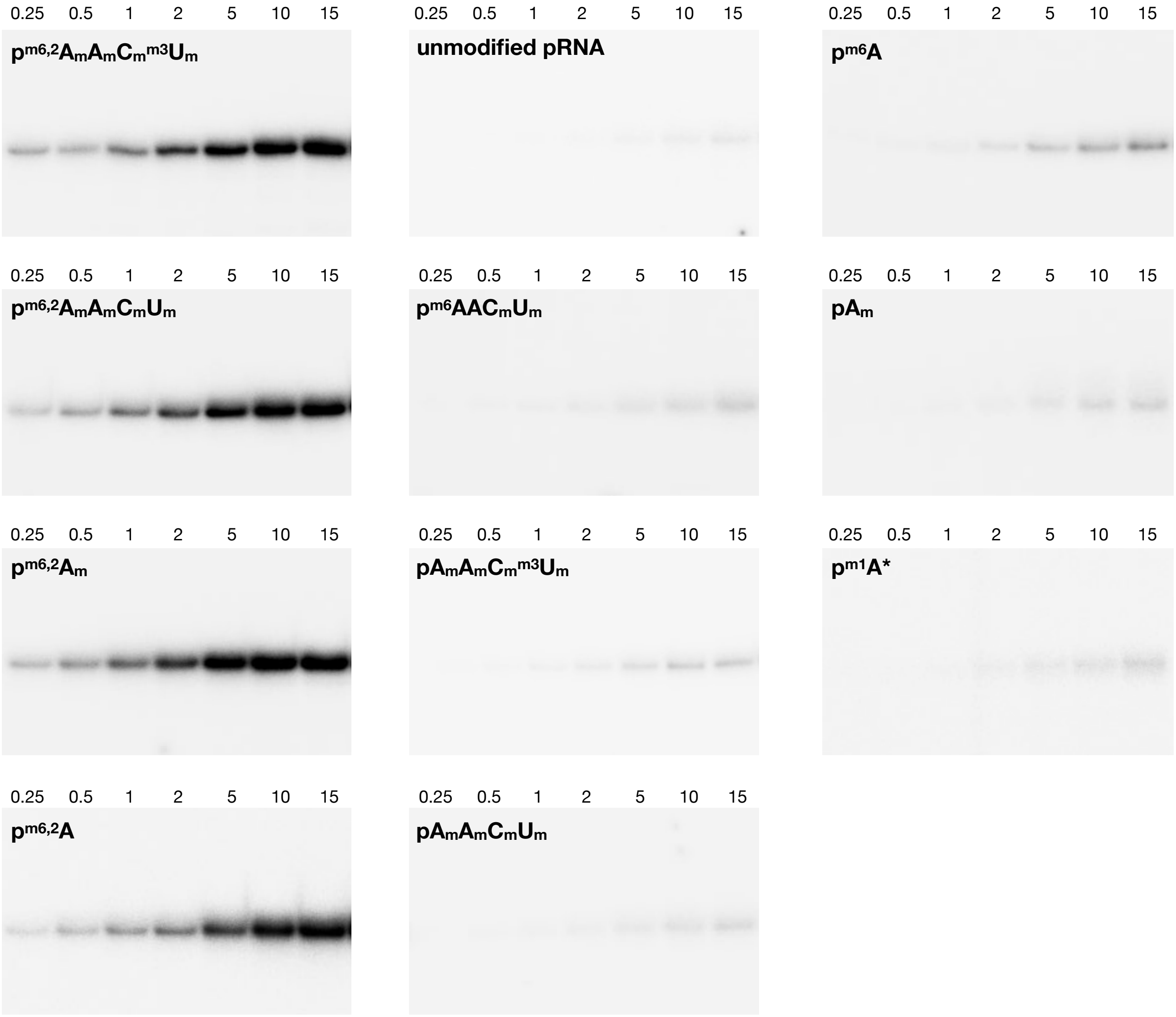

Supplementary Figure S2. TbCe1 RNA kinase assay. Representative gels for data in Figure 1B. RNA kinase assay (100 µl) containing 50 ng of TbCe1, 1 µM of $[\gamma\text{-}^{32}P]$ ATP, and either 100 nM of modified or unmodified pRNA as indicated were incubated at 27°C. An aliquot (10 µl) was withdrawn at the time (min) indicated, and the products were separated on an 18% Urea-PAGE. Gels were scanned by PhosphorImager. Representative gels are shown. *For $p^{m1}A$ RNA gel, the intensity of the image was adjusted to detect the products (adjusted image level set to 60%) on Apple Pages 14 provided on Mac OS version 14.41.

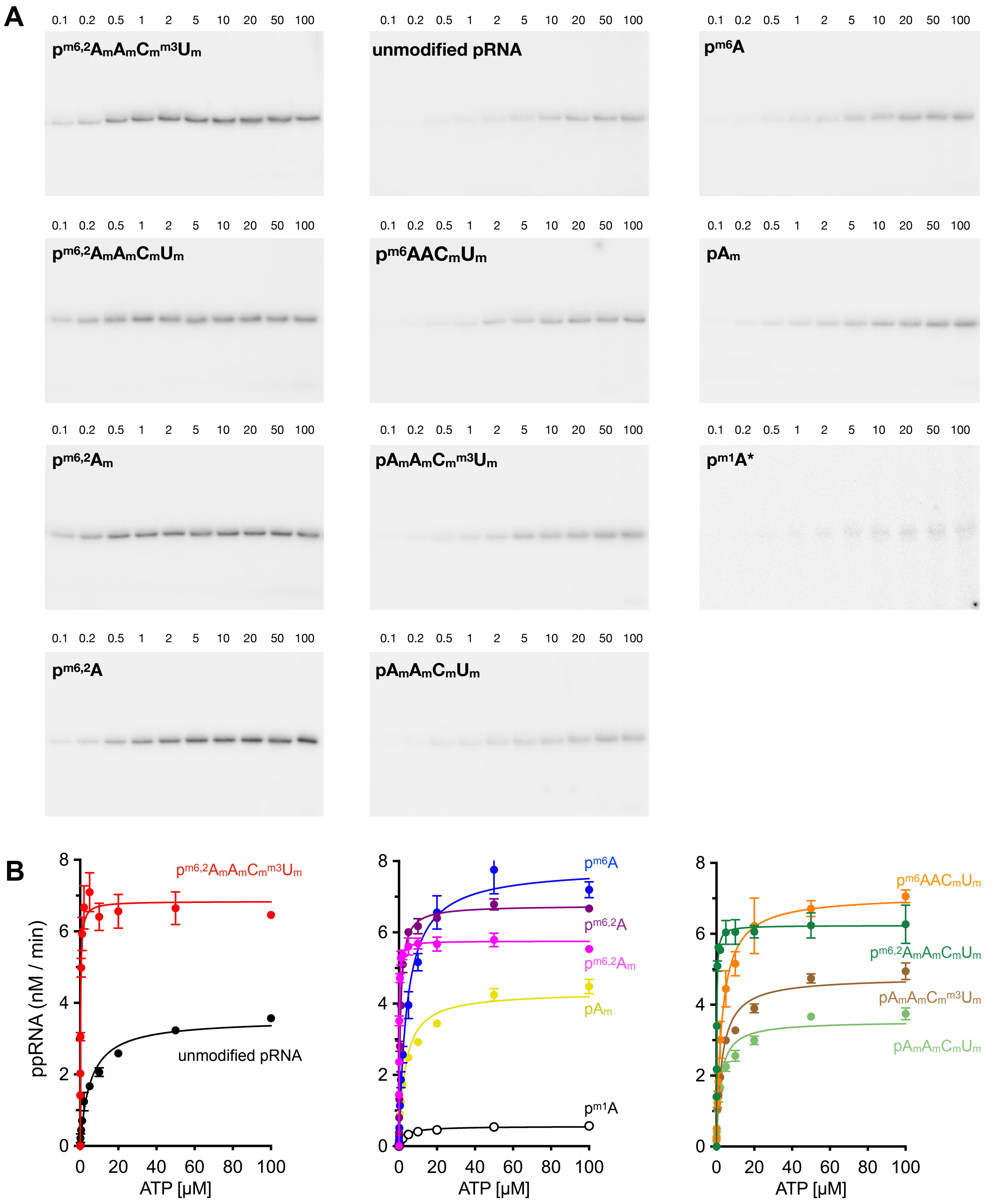

Supplementary Figure S3. TbCe1 RNA kinase assay on modified pRNAs at various ATP concentration. Representative gels for data in Figure 1C. Standard RNA kinase assay (10 μl) contained 100 nM of either modified or unmodified pRNA, 20 ng of TbCe1, and $[\gamma\text{-}^{32}P]$ ATP (μM) as indicated. Samples were separated on a denaturing 18% PAGE and scanned by PhosphorImager. (A) Representative gels are shown with ATP (μM) concentrations specified above the lanes. *For $p^{m1}A$ RNA gel, the intensity of the image was adjusted to detect the products (adjusted image level set to 90%) on Apple Pages 14 provided on Mac OS version 14.41. (B) The yield of the ppRNA product was plotted as a function of ATP concentration. See Figure 1C for ATP concentration between 0.1 - 10 μM.

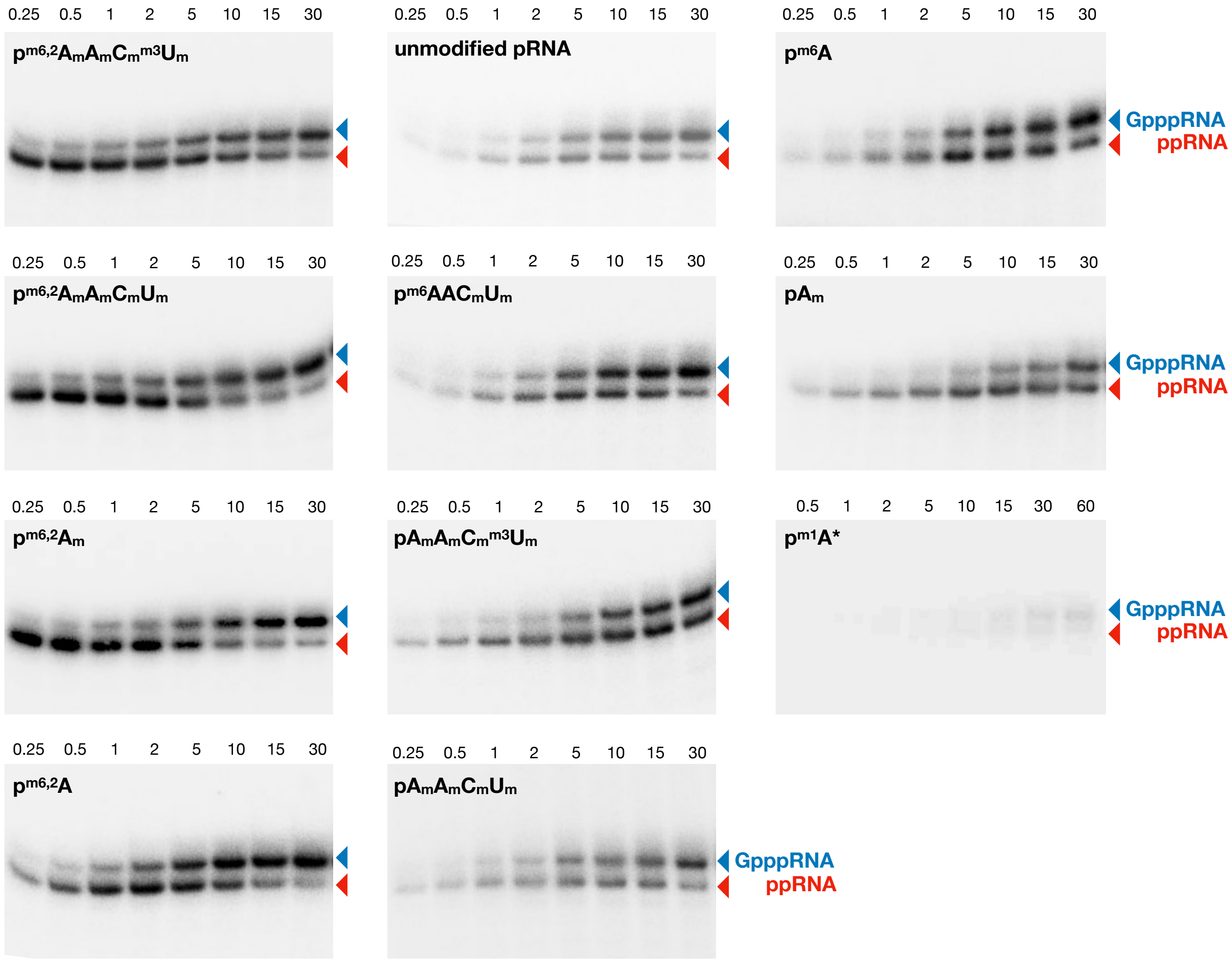

Supplementary Figure S4. Kinetics analysis of GpppRNA formation on modified and unmodified pRNA. Representative gels for data in Figure 2. The reaction mixture (100 µl) contained 50 mM Tris-HCl pH 8.5, 1 mM dithiothreitol, 0.5 mM $MgCl_2$, 500 ng of TbCe1, 10 µM $[\gamma\text{-}^{32}P]$ ATP, 10 µM GTP with 100 nM of modified and unmodified pRNA. An aliquot (10 µl) was withdrawn at the indicated time (min), and products were separated on 18% Urea-PAGE. Positions of ^{32}P-labeled GpppRNA (blue arrow) and ppRNA intermediate (red arrow) are indicated. *For $p^{m1}A$ RNA gel, the intensity of the image was adjusted to detect the products (adjusted image level set to 80%) on Apple Pages 14 provided on Mac OS version 14.41.

A

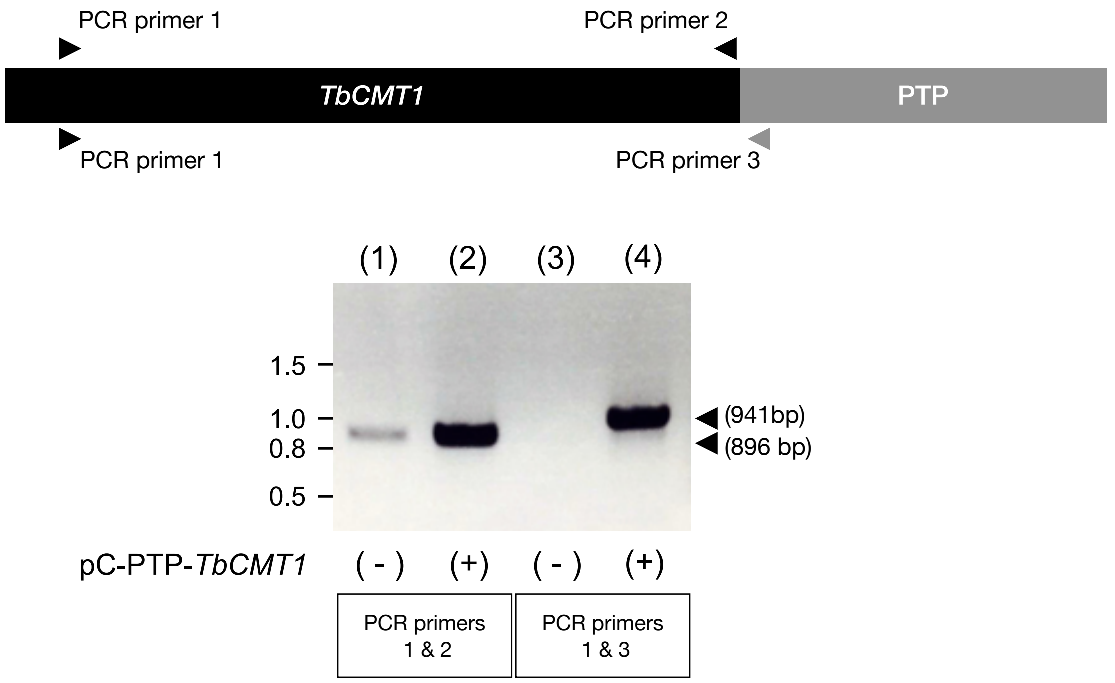

B

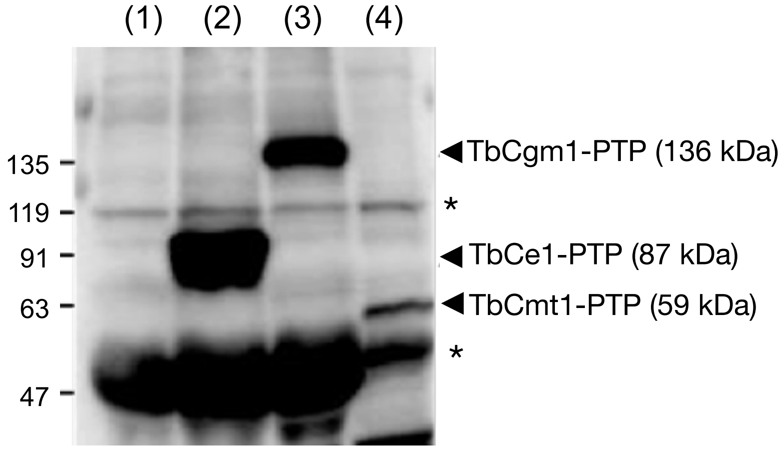

Supplementary Figure S5. Expression of PTP-tagged TbCmt1 fusion protein in *T.brucei*. (A) A protein C-TEV-Protein A (PTP) tag was introduced into the *TbCMT1* genomic loci by homologous recombination. The positions of three PCR primers are depicted. The lower panel shows an agarose gel electrophoresis of PCR products derived from cells transfected with pC-PTP-*TbCMT1* plasmid (+) or untransfected control (-). Positions of expected product sizes for pC-PTP-*TbCMT1* (941 bp) and wild-type (869 bp) are depicted. (B) Detection of PTP-tagged TbCmt1 fusion protein by Western blot. Cell lysates (25 µg) from untransfected *T. brucei brucei* Eatro 164 control (lane 1), TbCe1-PTP (lane 2), TbCgm1-PTP (lane 3) and TbCmt1-PTP (lane 4) were separated on SDS-PAGE and transferred to PVDF membrane. The PTP-tagged fusion proteins were detected by rabbit anti-Protein A antibody (Sigma) and horseradish peroxidase-conjugated secondary antibody. Position and predicted MW of PTP-fusion proteins are indicated. A nonspecific cross reacting ~50 kDa and ~120 kDa polypeptides are indicated by the asterisk.

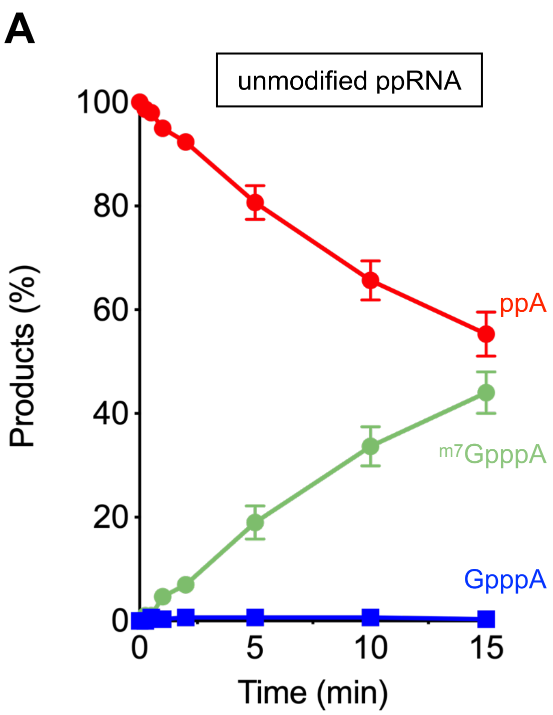

Supplementary Figure S6. Effect of 2'-O methylation on vaccinia cap enzyme. Reaction mixture (20 µl) contained 50 mM Tris-HCl (pH 8.5), 1 mM dithiothreitol, 2.5 mM $MgCl_2$, 50 µM GTP, 50 µM AdoMet, 4 units of vaccinia virus capping enzyme, and 1 pmol of either ^{32}P-labeled (A) unmodified ppRNA or (B) $ppA_mA_mC_mU_m$ RNA. The reaction was incubated at 37°C. Aliquot (2 µL) was withdrawn at each time point and samples were digested by nuclease P1. Aliquots of digested products were separated on TLC plate which was developed in 1 M formic acid and 0.5 M LiCl. Percent products [(ppA, GpppA and ^{m7}GpppA), or (ppA_m, $GpppA_m$, and $^{m7}GpppA_m$)] were plotted as a function of time. The data shown on the graph represent the average of three separate experiments with SE bars.
